# Waning Protection Against Severe COVID-19 Following Vaccination: A Longitudinal IPTW Analysis of Emergency Department Encounters

**DOI:** 10.3390/idr17060142

**Published:** 2025-11-13

**Authors:** Yuying Xing, Amit Bahl

**Affiliations:** 1 Corewell Health Research Institute, Corewell Health, Royal Oak, MI 48073, USA; yuying.xing@corewellhealth.org; 2 Department of Emergency Medicine, Corewell Health William Beaumont University Hospital, Royal Oak, MI 48073, USA; 3 Department of Emergency Medicine, Oakland University William Beaumont School of Medicine, Rochester, MI 48309, USA

**Keywords:** COVID-19, vaccine effectiveness, waning immunity, severe outcomes, inverse probability of treatment weighting (IPTW), emergency department

## Abstract

Background: The duration of protection that COVID-19 vaccination provides against severe outcomes remains uncertain. Accurately defining this timeframe is critical for informing effective vaccination policies and booster strategies. This investigation aimed to quantify the length and durability of vaccine-conferred protection against severe disease, delivering evidence to guide public health decision-making. Methods: We conducted a multi-site cohort study to evaluate the relationship between time since last COVID-19 vaccination and the risk of severe infection among emergency department (ED) patients with a principal diagnosis of COVID-19. Vaccination status was categorized by time since the last documented dose: unvaccinated, 0–6 months, 7–12 months, 13–18 months, and 19–24 months. The primary outcome was severe COVID-19, defined as ICU admission, mechanical ventilation, or in-hospital death. Inverse Probability of Treatment Weighting (IPTW) was used to adjust for baseline confounding based on age group, sex, race, comorbidity burden, immunocompromised status, and calendar time period (pre-2023 vs. post-2023). Cox proportional hazards models were used to estimate adjusted hazard ratios (aHRs) for each vaccination interval compared to unvaccinated patients. Results: Between 1 December 2021, and 20 July 2024, 42,124 ED encounters were included in the analysis. In IPTW-weighted models, vaccination within 0–6 months (aHR 0.73, 95% CI 0.64–0.83), 7–12 months (aHR 0.72, 95% CI 0.64–0.82), and 13–18 months (aHR 0.67, 95% CI 0.57–0.79) was associated with a significantly reduced risk of severe outcomes. However, no significant protection was observed at 19–24 months (aHR 0.95, 95% CI 0.80–1.14). In age-stratified analyses, protection persisted longer in individuals aged ≥65 years than in those aged 50–64. Older age, male sex, comorbidities, and immunocompromised status were also associated with increased risk. Conclusions: COVID-19 vaccination provides sustained protection against severe outcomes for up to 18 months, after which effectiveness declines substantially. These findings support booster dose strategies based on time since last vaccination and targeted prioritization for high-risk populations.

## 1. Introduction

Coronavirus disease 2019 (COVID-19), caused by the novel severe acute respiratory syndrome coronavirus 2 (SARS-CoV-2), has been a leading cause of global morbidity and mortality since its emergence in late 2019 [1,2]. The early phases of the pandemic were characterized by high rates of hospitalization, intensive care unit (ICU) admission, mechanical ventilation, and death, particularly among older adults and individuals with comorbid conditions. However, over time, population-level outcomes improved, driven by a combination of increased clinical experience, evolving therapeutic strategies, and, most significantly, widespread implementation of vaccination programs [3,4].

COVID-19 vaccines have consistently demonstrated strong protection against severe disease, hospitalization, and death across multiple studies and real-world datasets [5,6]. Vaccination has been particularly impactful in high-risk populations, mitigating the worst outcomes even amidst the emergence of new viral variants [7,8]. Since the beginning of the pandemic, annual vaccination campaigns have been implemented across regions, reflecting ongoing adaptation of vaccine formulations to circulating variants and evolving public health recommendations. These efforts underscore the dynamic nature of COVID-19 vaccine policy and the importance of maintaining population immunity over time [9,10].

Despite recommendations for annual COVID-19 vaccination, a substantial proportion of individuals who initially received the primary vaccine series or early booster doses have not remained up to date. As of late 2024, only about 17.9% of U.S. adults had received the updated COVID-19 vaccine for the 2024–2025 season, despite CDC recommendations for annual vaccination for everyone over six months of age [3,11]. This lapse in adherence may have important clinical implications, especially among hospitalized patients for whom protection from severe disease is paramount. While the initial effectiveness of COVID-19 vaccines against severe illness is well established, the durability of protection over longer intervals remains uncertain. Most real-world studies measure vaccine effectiveness (VE) within 6–12 months following vaccination, leaving a critical gap in understanding the longevity of protection and the timing of potential waning.

The objective of this study is to evaluate the relationship between the time elapsed since the last COVID-19 vaccination and the risk of severe outcomes among ED patients. Leveraging real-world data, we aim to gain insights into how waning immunity affects disease severity, guiding strategies for optimal vaccine timing and coverage.

## 2. Methods

### 2.1. Study Design

This was a retrospective, observational study conducted across multiple sites, focusing on patients who presented to the emergency department (ED) with confirmed acute COVID-19 infection. The study included data from eight hospitals within southeastern Michigan, encompassing a range of healthcare settings from small community hospitals to a large academic tertiary center.

### 2.2. Participant Selection

Eligible participants were patients who presented to one of the eight Corewell Health East emergency departments between 1 December 2021, and 20 July 2024, and received a primary diagnosis of COVID-19. There was no age restriction in the selection of participants; all patients presenting with a principal diagnosis of COVID-19 were eligible regardless of age. The Corewell Health Institutional Review Board approved the study protocol. Given its retrospective design, the requirement for written informed consent was waived. All procedures were carried out in accordance with institutional policies for human subjects research and the ethical standards outlined in the Declaration of Helsinki.

### 2.3. Data Sources

Patient data were obtained from the Epic electronic health record (EHR) system (February 2025 version, Epic Systems Corporation, Verona, WI, USA), capturing a range of demographic, clinical, laboratory, and outcome variables. These included patient age, sex, ethnicity, medical history, COVID-19 vaccination status, initial vital signs, hospital treatments, and major clinical outcomes such as ICU admission, mechanical ventilation, mortality, and length of hospital stay. Comorbidity burden was quantified using the Elixhauser Comorbidity Index as defined by the Agency for Healthcare Research and Quality (AHRQ) [12,13], and missing observations were treated as a separate category in all analyses to preserve sample size and minimize bias. Immunocompromised status was determined using ICD-10 codes consistent with the AHRQ criteria, including autoimmune disorders, organ transplant history, chronic illnesses, nutritional deficiencies, genetic conditions, and HIV. Vaccination records were confirmed using the institution’s EHR system, which is integrated with the Michigan Care Improvement Registry (MCIR). This linkage enabled comprehensive verification of COVID-19 vaccination history, including immunizations received outside the Corewell Health system, with detailed documentation of vaccine types and administration date. This approach significantly reduced missing data related to vaccination status. Encounters without documented vaccination in either the EHR or MCIR were classified as unvaccinated.

Measures Vaccination status was defined by the receipt of any COVID-19 vaccine since the onset of the pandemic. Patients were further stratified based on the time elapsed between their most recent vaccine dose and their COVID-19 diagnosis requiring emergency care, grouped in six-month intervals. Those whose last vaccine dose was administered more than 24 months prior to the index visit were excluded due to small sample size and limited interpretability.

We also included a calendar time variable to account for differences in circulating SARS-CoV-2 variants. Encounters were categorized as occurring in the pre-2023 period (1 December 2021–31 December 2022) or the post-2023 period (1 January 2023–20 July 2024). This classification was based on the timing of major Omicron subvariant waves and subsequent shifts in dominant lineages in the USA [14].

### 2.4. Outcomes

The primary aim of this study was to evaluate whether the timing of COVID-19 vaccination influenced the risk of severe clinical outcomes occurring during the index encounter from the emergency department. Severe infection was defined as a composite outcome that included admission to the intensive care unit (ICU), need for mechanical ventilation, or in-hospital mortality. Time to severe infection was calculated from the time of hospital arrival to the occurrence of any of the composite events.

A pre-specified subgroup analysis was conducted by age group (50–64 years vs. ≥65 years) to examine whether the association between vaccination timing and severe outcomes varied by age. Younger age groups (<50 years) were not included in subgroup analyses because the incidence of severe COVID-19 in this population was low, limiting statistical power and interpretability. Focusing on higher-risk age groups allowed for more meaningful estimation of vaccine effectiveness across longer time intervals.

### 2.5. Statistical Analysis

Baseline characteristics were described across groups defined by time since last COVID-19 vaccination. Continuous variables were reported as means with standard deviations and compared using ANOVA tests. Categorical variables were summarized as counts and percentages and compared using Chi-square tests.

To adjust for confounding, inverse probability of treatment weighting (IPTW) was performed using a multinomial logistic regression model, appropriate for the multi-level exposure (time since last vaccination). The propensity score model included age group, sex, race, Elixhauser comorbidity index, immunocompromised status, and calendar time period (pre-2023 vs. post-2023). IPTW was used to create a pseudo-population in which the distribution of covariates is independent of vaccination status, minimizing bias from confounding and improving the validity of causal effect estimates [15,16]. After weighting, covariate balance was assessed using standardized mean differences (SMDs), with values below 0.1 considered acceptable (Supplementary Figure S1).

Subsequently, Cox proportional hazards regression was used to examine the association between vaccination timing and the risk of severe infection. Two modeling approaches were implemented: multivariable Cox models, adjusted for age, sex, race, comorbidities, immunocompromised status, and time period; and IPTW Cox models, which incorporated the inverse probability weights and included age group as an additional covariate. Although age group was included in the original propensity score model, it remained imbalanced after weighting (SMD > 0.1; Supplementary Figure S1); therefore, it was retained in the IPTW Cox models to reduce residual confounding and improve robustness. We assessed the proportional hazards assumption for IPTW Cox model using Schoenfeld residuals. The assumption was met for the main variable, vaccine group, but was violated by the covariate age group. To address this violation and ensure model validity, we stratified the Cox model by age group.

Subgroup analyses stratified by age groups (50–64 and 65+ years) were also conducted to explore potential effect modification. All models were adjusted for the same covariates, excluding the stratification variable.

Hazard ratios (HRs) were reported with corresponding 95% confidence intervals (CIs) and *p*-values for the Cox proportional hazards regression analysis. All statistical tests were two-sided, and statistical significance was determined using a *p*-value threshold of less than 0.05. Analyses were performed using R-4.3.1 (R Foundation for Statistical Computing, Vienna, Austria). 

## 3. Results

**Figure 1:**
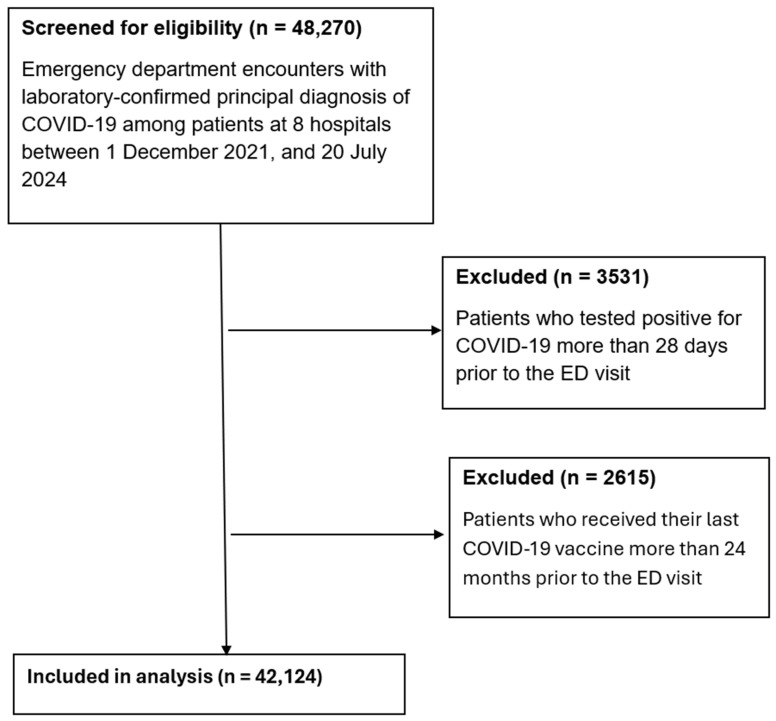
Flow figure of inclusion and exclusion criteria. Flow figure demonstrating total number encounters screened for eligibility, number of excluded encounters, and final number of encounters included in the analysis.

A total of 48,270 emergency department encounters with principal diagnosis of COVID-19 were screened for eligibility between 1 December 2021, and 20 July 2024 (Figure 1). The age of participants ranged from less than 1 year to over 100 years. After excluding 2615 encounters where the last COVID-19 vaccine was administered more than 24 months prior to the visit and 3531 encounters where patients tested positive more than 28 days before presentation, 42,124 encounters were included in the final analysis.

Baseline characteristics stratified by time since last COVID-19 vaccination are shown in Table 1. Prior to weighting, there were significant differences in age distribution, sex, race, comorbidity burden, immunocompromised status, and time period across vaccination groups (all *p*-values < 0.001). For example, patients vaccinated within the past 6 months were older on average (mean age: 61.3 years) compared to unvaccinated patients (mean age: 35.4 years). After applying IPTW, covariate balance improved substantially across groups. Most variables achieved SMD < 0.1, although age group remained imbalanced with SMD > 0.1 (Supplementary Figure S1), and was therefore included as a covariate in subsequent IPTW Cox models.

**Figure 2:**
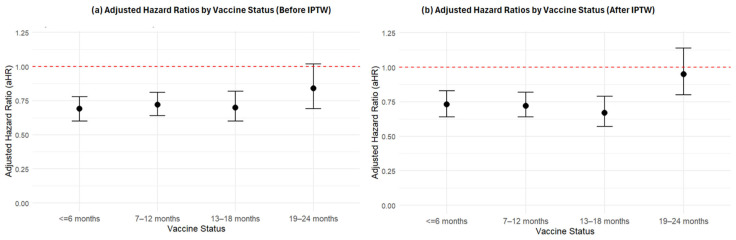
Adjusted Hazard Ratios by Vaccine Status Using Cox Proportional Hazards Models. Abbreviations: aHR = adjusted hazard ratio. (**a**) Multivariable Cox model before IPTW adjusted for age, sex, race, comorbidities, immunocompromised status, and time period. (**b**) IPTW Cox model adjusted for age.

Results from Cox proportional hazards regression models are presented in Table 2 and Figure 2. In both the before and after IPTW models, COVID-19 vaccination was associated with a significantly lower risk of severe infection compared to no vaccination. Importantly, the protective effect remained relatively stable through the first 18 months after vaccination. In the IPTW model, individuals vaccinated within 6 months had a 27% lower hazard of severe infection (aHR: 0.73; 95% CI: 0.64–0.83; *p* < 0.001), and those vaccinated 7–12 months and 13–18 months prior showed similar levels of protection (aHR: 0.72; 95% CI: 0.64–0.82; *p* < 0.001; and aHR: 0.67; 95% CI: 0.57–0.79; *p* < 0.001, respectively). However, the protective effect declined sharply beyond 18 months, with the 19–24 months group showing no statistically significant reduction in risk compared to the unvaccinated group (aHR: 0.95; 95% CI: 0.80–1.14; *p* = 0.711). This pattern suggests that vaccine-associated protection against severe infection remains stable up to 18 months, but wanes significantly thereafter.

**Figure 3:**
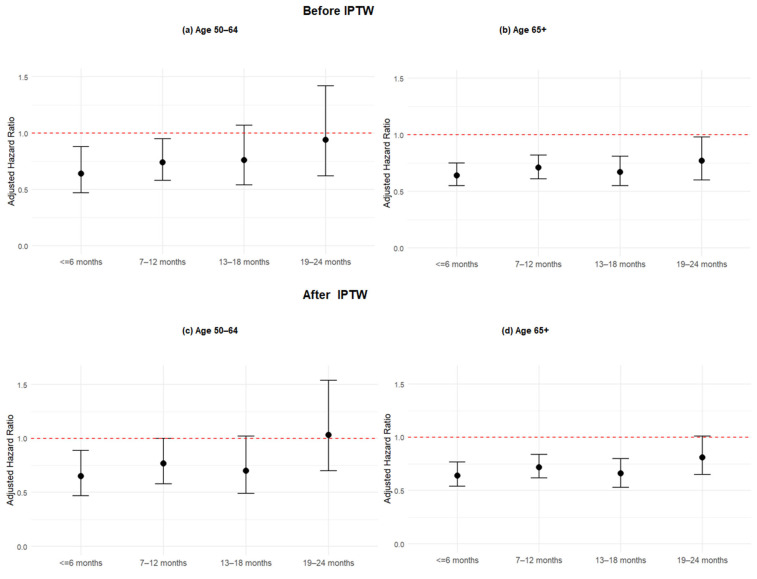
Multivariable Cox Proportional Hazards Regressions for Risk of Severe Infection Stratified by Age Group. Each panel shows the results of multivariable and IPTW Cox proportional hazards regression models for a specific age group. Hazard ratios (HRs) and 95% confidence intervals (CIs) are shown. Multivariable Cox models are adjusted for sex, race, comorbidities, immunocompromised status and time period.

In subgroup analyses stratified by age groups using IPTW Cox models (Supplementary Table S1 and Figure 3), the protective effect of COVID-19 vaccination declined over time, particularly beyond 18 months. Among patients aged 65 years and older, those vaccinated 13–18 months prior to their emergency department visit continued to exhibit a significantly reduced risk of severe outcomes compared to the unvaccinated group (aHR: 0.66; 95% CI: 0.53–0.80; *p* < 0.001). In contrast, the 50–64 age group showed a weaker and non-significant association at the same interval (aHR: 0.70; 95% CI: 0.49–1.02; *p* = 0.053), suggesting that the protective effect of prior vaccination may wane more rapidly in the 50–64 group or that the effect is less consistent in this subgroup. By 19–24 months, vaccine-associated protection was no longer statistically significant in either age group (65+: aHR: 0.81; 95% CI: 0.65–1.01; 50–64: aHR: 1.03; 95% CI: 0.70–1.54). These findings indicate that while some protection persists up to 18 months in older adults, the decline is more pronounced in the 50–64 age group, highlighting potential differences in long-term immune response and reinforcing the importance of timely booster vaccination in both groups.

## 4. Discussion

In this large, retrospective cohort study of emergency department encounters with principal diagnosis of COVID-19, we found that prior COVID-19 vaccination was associated with a significantly lower risk of severe infection. Notably, this protective effect remained relatively stable through the first 18 months after the most recent vaccine dose. However, protection declined sharply beyond that period, with no significant benefit observed in individuals vaccinated more than 18 months prior to presentation. These findings indicate a clear threshold in the durability of vaccine-conferred protection and provide real-world evidence supporting time-based considerations for revaccination.

Subgroup analyses stratified by age further revealed differences in the persistence of protection. Among individuals aged 65 years and older, vaccine effectiveness remained significant up to 18 months post-vaccination. In contrast, the 50–64 age group showed less consistent protection during the same timeframe and no significant benefit beyond 18 months. These results align with immunologic studies suggesting age-related differences in immune response and durability following COVID-19 vaccination [17,18]. The data also support the need for targeted booster strategies that prioritize both timing and high-risk subpopulations [19].

Our findings align with previous research demonstrating that vaccine effectiveness (VE) against severe COVID-19 outcomes remains strong and durable. A recent systematic review and meta-regression by Feikin et al. found that VE against severe COVID-19 disease declined by only 10 percentage points over six months, with most estimates consistently above 70% [5]. Similarly, Andrews et al. reported sustained high VE against hospitalization and death for at least 20 weeks post-vaccination, with minimal evidence of waning [20]. Our study extends this body of work by evaluating VE over a longer time frame—up to two years—and reveals that protection against severe outcomes remains remarkably stable for 18 months before experiencing a sharp decline. While VE against infections and milder disease may gradually decrease, as noted by Wu et al. [21], our findings underscore the resilience of protection against severe disease, reinforcing the notion of sustained immunity for up to 18 months before significant waning occurs. These data highlight the importance of optimizing vaccination strategies that maximize protection while considering the potential for adverse reactions and the overall risk–benefit balance for specific patient populations [22,23,24].

While time-to-event analysis allowed for robust modeling of risk using Cox regression, we acknowledge that the time from hospital arrival to severe outcome may be influenced by factors unrelated to vaccination status. Delays in care-seeking, pre-hospital disease progression, and institutional treatment protocols could all affect the timing of clinical deterioration. Therefore, the hazard ratios should be interpreted as relative risks of severe outcomes rather than precise indicators of disease progression speed.

## 5. Limitations

This study has several limitations. First, residual confounding is possible despite the use of IPTW and multivariable adjustment. Second, although we excluded patients tested positive more than 28 days before presentation, incomplete or unrecorded infection history may still introduce bias. We were unable to fully account for prior natural infection, which may have contributed to observed protection, particularly among vaccinated individuals. This potential for hybrid immunity could influence the magnitude of vaccine-associated protection and should be considered when interpreting the results. Third, while we used calendar time period (pre-2023 vs. post-2023) to control for differences in circulating variants, this approach may not fully capture the complexity of variant-specific dynamics. Fourth, the use of large electronic health record (EHR) datasets, while enabling inclusion of a broad and diverse patient population, carries inherent limitations related to missing, incomplete, or inaccurate data. These data quality issues may have led to misclassification bias, particularly among immunocompromised patients, where identification relied on ICD-10 coding rather than direct clinical validation. Additionally, the definition of severe infection—based on ICU admission, mechanical ventilation, or in-hospital mortality—may not encompass all clinically meaningful severe outcomes and could be influenced by hospital-level practices or capacity constraints. Finally, vaccine type and number of doses were not stratified in this analysis, which may influence the generalizability of results to specific vaccine platforms.

## 6. Conclusions

In this large real-world study of emergency department encounters with COVID-19, we found that prior vaccination was associated with sustained protection against severe outcomes for up to 18 months. The protective effect remained stable throughout the first 18 months following the last dose but declined significantly beyond that period, with no statistically significant benefit observed at 19–24 months. Age-stratified analysis showed that older adults (≥65 years) retained stronger and more durable protection than those aged 50–64, though both groups experienced waning effectiveness after 18 months. These findings underscore the importance of incorporating time since last vaccination into risk assessment and booster policy planning. Timely revaccination—particularly for older adults, individuals with multiple comorbidities, and immunocompromised populations—may be critical to maintaining protection against severe COVID-19 outcomes.

## Figures and Tables

**Table 1:** Patient characteristics.

	COVID Vaccination Status											
	Before IPTW						After IPTW					
	Unvaccinated	<=6 Months	7–12 Months	13–18 Months	19–24 Months	*p* Value	Unvaccinated	<=6 Months	7–12 Months	13–18 Months	19–24 Months	SMD
N	21,742	5920	7835	4235	2391		21,765.2	5753.3	7251.3	3916.8	2093.3	
Age, years						<0.001 ^1^						0.190
Mean (SD)	35.4 (25.7)	61.3 (22.4)	59.6 (21.0)	59.9 (21.3)	59.2 (21.1)		46.75 (26.72)	49.59 (25.65)	52.13 (23.69)	51.75 (23.53)	57.14 (22.94)	
Age Group						<0.001 ^2^						0.192
<18	6064 (27.9%)	283 (4.8%)	212 (2.7%)	102 (2.4%)	52 (2.2%)		3463.6 (15.9%)	822.6 (14.3%)	718.3 (9.9%)	338.7 (8.6%)	142.5 (6.8%)	
18–49	9016 (41.5%)	1280 (21.6%)	2140 (27.3%)	1180 (27.9%)	697 (29.2%)		7389.8 (34.0%)	1928.9 (33.5%)	2562.2 (35.3%)	1482.1 (37.8%)	621.0 (29.7%)	
50–64	3213 (14.8%)	1186 (20.0%)	1847 (23.6%)	945 (22.3%)	563 (23.5%)		4039.6 (18.6%)	1091.5 (19.0%)	1428.7 (19.7%)	767.8 (19.6%)	407.6 (19.5%)	
65+	3449 (15.9%)	3171 (53.6%)	3637 (46.4%)	2008 (47.4%)	1079 (45.1%)		6872.3 (31.6%)	1910.3 (33.2%)	2542.1 (35.1%)	1328.2 (33.9%)	922.1 (44.1%)	
Sex						<0.001 ^2^						0.017
Female	11,866 (54.6%)	3324 (56.1%)	4556 (58.1%)	2578 (60.9%)	1501 (62.8%)		12,567.8 (57.7%)	3344.8 (58.1%)	4307.2 (59.4%)	2254.2 (57.6%)	1208.8 (57.7%)	
Male	9876 (45.4%)	2596 (43.9%)	3280 (41.9%)	1657 (39.1%)	890 (37.2%)		9197.5 (42.3%)	2408.5 (41.9%)	2944.1 (40.6%)	1662.6 (42.4%)	884.5 (42.3%)	
Race						<0.001 ^2^						0.060
Black Race	9053 (41.6%)	1641 (27.7%)	2216 (28.3%)	1169 (27.6%)	763 (31.9%)		7557.9 (34.7%)	1931.8 (33.6%)	2398.4 (33.1%)	1320.1 (33.7%)	605.0 (28.9%)	
White Race	11,082 (51.0%)	3918 (66.2%)	5057 (64.5%)	2783 (65.7%)	1475 (61.7%)		12,628.5 (58.0%)	3383.1 (58.8%)	4336.8 (59.8%)	2324.3 (59.3%)	1345.6 (64.3%)	
Other	1607 (7.4%)	361 (6.1%)	563 (7.2%)	283 (6.7%)	153 (6.4%)		1578.8 (7.3%)	438.5 (7.6%)	516.1 (7.1%)	272.4 (7.0%)	142.7 (6.8%)	
Elixhauser Comorbidity Index						<0.001 ^2^						0.093
<0	3190 (14.7%)	1286 (21.7%)	1809 (23.1%)	959 (22.6%)	544 (22.8%)		4033.0 (18.5%)	1106.3 (19.2%)	1465.8 (20.2%)	775.3 (19.8%)	439.6 (21.0%)	
0	7696 (35.4%)	1330 (22.5%)	1765 (22.5%)	903 (21.3%)	548 (22.9%)		6287.7 (28.9%)	1617.3 (28.1%)	1939.3 (26.7%)	1018.1 (26.0%)	506.2 (24.2%)	
1 to 4	1172 (5.4%)	508 (8.6%)	696 (8.9%)	376 (8.9%)	211 (8.8%)		1544.5 (7.1%)	421.2 (7.3%)	554.5 (7.6%)	297.9 (7.6%)	167.5 (8.0%)	
>=5	3349 (15.4%)	2087 (35.3%)	2547 (32.5%)	1466 (34.6%)	812 (34.0%)		5289.4 (24.3%)	1443.8 (25.1%)	1931.0 (26.6%)	1017.3 (26.0%)	629.9 (30.1%)	
NA	6335 (29.1%)	709 (12.0%)	1019 (13.0%)	531 (12.5%)	276 (11.5%)		4610.6 (21.2%)	1164.7 (20.2%)	1360.6 (18.8%)	808.2 (20.6%)	350.1 (16.7%)	
Immunocompromised Patient						<0.001 ^2^						0.024
No	20,254 (93.2%)	4929 (83.3%)	6669 (85.1%)	3638 (85.9%)	2078 (86.9%)		19,354.9 (88.9%)	5096.8 (88.6%)	6403.0 (88.3%)	3472.5 (88.7%)	1825.3 (87.2%)	
Yes	1488 (6.8%)	991 (16.7%)	1167 (14.9%)	597 (14.1%)	313 (13.1%)		2410.3 (11.1%)	656.5 (11.4%)	848.3 (11.7%)	444.3 (11.3%)	267.9 (12.8%)	
Time Group						<0.001 ^2^						0.100
Pre-2023	16,135 (74.2%)	4618 (78.0%)	6487 (82.8%)	2211 (52.2%)	525 (22.0%)		15,758.4 (72.4%)	4337.7 (75.4%)	5334.6 (73.6%)	2729.8 (69.7%)	1381.1 (66.0%)	
Post-2023	5607 (25.8%)	1302 (22.0%)	1349 (17.2%)	2024 (47.8%)	1866 (78.0%)		6006.8 (27.6%)	1415.6 (24.6%)	1916.7 (26.4%)	1187.0 (30.3%)	712.1 (34.0%)	
Severe Outcome						<0.001 ^2^						
No	20,901 (96.1%)	5576 (94.2%)	7397 (94.4%)	4019 (94.9%)	2262 (94.6%)							
Yes	841 (3.9%)	344 (5.8%)	439 (5.6%)	216 (5.1%)	129 (5.4%)							

Abbreviations: SMD = Standardized Mean Difference. ^1^ ANOVA test. ^2^ Chi-square test. NA = missing data for Elixhauser comorbidity variable.

**Table 2:** Multivariable and IPTW Cox proportional hazards regressions for risk of severe infection stratified by age group.

Characteristics	Before IPTW			After IPTW		
	aHR	95% CI	*p*-Value	aHR	95% CI	*p*-Value
Vaccine Status						
Unvaccinated						
<=6 months	0.69	0.60, 0.78	<0.001	0.73	0.64, 0.83	<0.001
7–12 months	0.72	0.64, 0.81	<0.001	0.72	0.64, 0.82	<0.001
13–18 months	0.70	0.60, 0.82	<0.001	0.67	0.57, 0.79	<0.001
19–24 months	0.84	0.69, 1.02	0.087	0.95	0.80, 1.14	0.711
Age						
<18						
18–49	1.35	1.03, 1.75	0.027			
50–64	2.69	2.08, 3.48	<0.001			
65+	3.42	2.66, 4.40	<0.001			
Sex						
Female						
Male	1.35	1.23, 1.47	<0.001			
Race Group						
Black or African American						
White or Caucasian	1.23	1.11, 1.38	<0.001			
Other	1.7	1.41, 2.05	<0.001			
Elixhauser Comorbidity						
<0						
0	1.08	0.92, 1.27	0.349			
1 to 4	1.41	1.17, 1.71	<0.001			
>=5	1.53	1.34, 1.76	<0.001			
NA	1.11	0.93, 1.34	0.258			
Immunocompromised Patient						
No						
Yes	1.35	1.21, 1.51	<0.001			
Time Group						
Pre-2023						
Post-2023	0.85	0.76, 0.95	0.004			

Abbreviations: aHR = adjusted hazard ratio, CI = confidence interval. Multivariable Cox model adjusted for sex, age, race, comorbidities, immunocompromised status and time period. IPTW Cox model stratified by age. NA = missing data for Elixhauser comorbidity variable.

## Data Availability

The datasets generated and analyzed during the current study are not publicly available due to privacy and institutional restrictions but are available from the Principal Investigator on reasonable request.

## Supplementary Materials

The following supporting information can be downloaded at: https://www.mdpi.com/article/10.3390/idr17060142/s1, Table S1: Multivariable and IPTW Cox proportional hazards regressions for risk of severe infection stratified by age group; Figure S1: Standardized mean differences of different cohorts.
