# Supplementary material for: Waning Protection Against Severe COVID-19 Following Vaccination: A Longitudinal IPTW Analysis of Emergency Department Encounters
Source: Infect Dis Rep. 2025 Nov 13;17(6):142. doi: 10.3390/idr17060142 (PMC12641896; doi:10.3390/idr17060142)
Supplement: Supplementary file 1 [file idr-17-00142-s001.zip › idr-3888408-supplementary-Final Proofreading Done.pdf]

Supplementary Table S1. Multivariable and IPTW Cox proportional hazards regressions for risk of severe infection stratified by age group

| | Before IPTW | | | | | | After IPTW | | | | | |
|---|---|---|---|---|---|---|---|---|---|---|---|---|
| | Age 50-64 | | | Age 65+ | | | Age 50-64 | | | Age 65+ | | |
| **Characteristics** | **aHR** | **95% CI** | **P-value** | **aHR** | **95% CI** | **P-value** | **aHR** | **95% CI** | **P-value** | **aHR** | **95% CI** | **P-value** |
| Vaccine Status | | | | | | | | | | | | |
| Unvaccinated | | | | | | | | | | | | |
| <=6 months | 0.64 | 0.47, 0.88 | 0.005 | 0.64 | 0.55, 0.75 | <0.001 | 0.65 | 0.47, 0.89 | 0.007 | 0.64 | 0.54, 0.77 | <0.001 |
| 7-12 months | 0.74 | 0.58, 0.95 | 0.020 | 0.71 | 0.61, 0.82 | <0.001 | 0.77 | 0.58, 1.00 | 0.041 | 0.72 | 0.62, 0.84 | <0.001 |
| 13-18 months | 0.76 | 0.54, 1.07 | 0.110 | 0.67 | 0.55, 0.81 | <0.001 | 0.70 | 0.49, 1.02 | 0.053 | 0.66 | 0.53, 0.80 | <0.001 |
| 19-24 months | 0.94 | 0.62, 1.42 | 0.754 | 0.77 | 0.60, 0.98 | 0.032 | 1.03 | 0.70, 1.54 | 0.899 | 0.81 | 0.65, 1.01 | 0.200 |
| Sex | | | | | | | | | | | | |
| Female | | | | | | | | | | | | |
| Male | 1.46 | 1.20, 1.77 | <0.001 | 1.29 | 1.15, 1.44 | <0.001 | | | | | | |
| Race Group | | | | | | | | | | | | |
| Black or African American | | | | | | | | | | | | |
| White or Caucasian | 1.32 | 1.06, 1.65 | 0.012 | 1.18 | 1.01, 1.37 | 0.033 | | | | | | |
| Other | 1.16 | 0.73, 1.85 | 0.540 | 1.96 | 1.55, 2.48 | <0.001 | | | | | | |
| Elixhauser Comorbidity | | | | | | | | | | | | |
| <0 | — | — | | — | — | | | | | | | |
| 0 | 1.17 | 0.87, 1.57 | 0.307 | 1.18 | 0.95, 1.46 | 0.135 | | | | | | |
| 1 to 4 | 1.28 | 0.87, 1.87 | 0.207 | 1.33 | 1.04, 1.70 | 0.022 | | | | | | |
| >=5 | 1.31 | 1.00, 1.71 | 0.052 | 1.55 | 1.30, 1.85 | <0.001 | | | | | | |
| NA | 0.78 | 0.53, 1.14 | 0.202 | 1.38 | 1.07, 1.78 | 0.013 | | | | | | |
| Immunocompromised Patient | | | | | | | | | | | | |
| No | | | | | | | | | | | | |
| Yes | 1.61 | 1.28, 2.02 | <0.001 | 1.22 | 1.06, 1.39 | 0.004 | | | | | | |
| Time Group | | | | | | | | | | | | |
| Pre-2023 | | | | | | | | | | | | |
| Post-2023 | 0.81 | 0.62, 1.05 | 0.118 | 0.89 | 0.78, 1.01 | 0.077 | | | | | | |

Abbreviations: aHR = adjusted hazard ratio, CI = confidence interval, NA = missing data for Elixhauser comorbidity variable.
Multivariable Cox model adjusted for sex, race, comorbidities, immunocompromised status and time period.

Supplementary Figure S1. Standardized mean differences of different cohorts

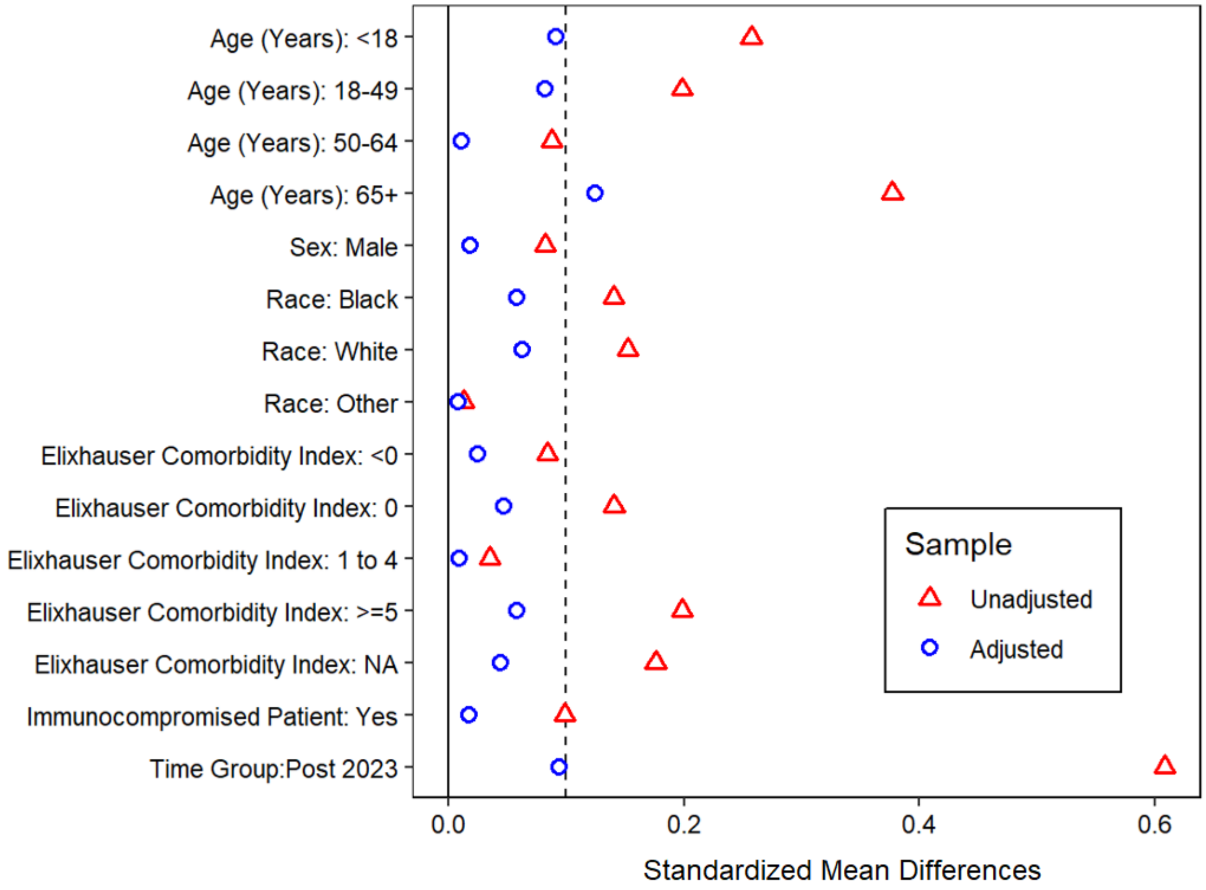


This figure displays the standardized mean differences (SMDs) for baseline covariates across COVID-19 vaccination groups before and after inverse probability of treatment weighting (IPTW). Lower SMD values indicate better covariate balance among groups. The horizontal reference line at 0.1 represents the conventional threshold for acceptable balance.
